# 

**Published:** 1884-02

**Authors:** 


### CONTENTS:

Art. I.—The Conservative Treatment of the Pulp. By James G. Palmer, D. D. S....433

Art. II.—Treatment of Fracture of the Jaw, (Continued.) By Thos. Brian Gunning, D. D. S..........443

Art. III.—A Résume of the Different Preparations of Gold Used for Filling Teeth. By George C. Brown..........448

Art. IV.—Odontological Society of Pennsylvania..........456

Art. V.—Tin Foil as a Filling..........460

Art. VI.—Effects of Malarial Poisoning on the Dental Pulp..........465

Art. VII.—Hardening Dental Rubbers in Steatite Without the Aid of Flasks..........469

### EDITORIAL, ETC.

Death in the Dental Chair..........471
The Maryland Dental Bill..........473
University of Maryland, Dental Department Annual Commencement..........474
Alumni Meeting..........474

### OBITUARY.

Dr. Robert Early..........475

Dr. Arthur C. Ford..........475

### BIBLIOGRAPHICAL.

Dental Medicine. By Ferdinand J. S. Gorgas, M. D., D. D. S..........476

### MONTHLY SUMMARY.

Danger—Carelessness..........478

Chromic Acid as a Caustic..........480

